# Imidacloprid poisoning in a young female: a case report

**DOI:** 10.1186/s13256-022-03742-8

**Published:** 2023-02-11

**Authors:** Om Prakash Bhatta, Sabita Chand, Hemant Chand, Ram Chandra Poudel, Ram Prasad Lamichhane, Abhi Kumar Singh, Nuwadatta Subedi

**Affiliations:** 1 Department of Emergency Medicine, Nova Hospital, Dhangadhi, Nepal; 2 Department of Emergency Medicine, Nepalgunj Medical College, Chisapani, Nepal; 3 Department of Internal Medicine, Nova Hospital, Dhangadhi, Nepal; 4 Department of Psychiatry, Nova Hospital, Dhangadhi, Nepal; 5 Department of Forensic Medicine and Toxicology, Gandaki Medical College, Pokhara, Nepal

**Keywords:** Imidacloprid, Neonicotinoids, CNS toxicity

## Abstract

**Background:**

Imidacloprid, a neonicotinoid insecticide, is widely used in agricultural settings. Consequently, cases of accidental and suicidal poisoning are increasingly seen in clinical practice. Although cases with varied clinical presentations and toxicological profiles have been reported, standard management principles are lacking.

**Case presentation:**

We present a case of Imidacloprid poisoning in a 25-year-old previously healthy indigenous Tamang female without a classic toxidrome requiring ventilatory support, complicated by a prolonged neuropsychiatric sequela.

**Conclusions:**

Although uncommonly reported, imidacloprid toxicity may lead to life-threatening complications and hence should be suspected in cases of unidentified poisoning with a relevant toxidrome. Vigilance on the part of treating physicians plays a crucial role in appropriate management.

## Background

Imidacloprid, a neonicotinoid insecticide, is widely used in agricultural crop protection and flea control worldwide owing to its lower toxicity than organophosphorus compounds [1]. Consequently, cases of accidental as well as suicidal poisoning are increasingly seen in clinical practice [2].

Although cases with varied clinical presentations and toxicological profiles have been reported, standard management principles are lacking.

It classically presents with nausea or vomiting, abdominal pain, drowsiness, headache, or dizziness, but some cases may be asymptomatic [1].

Despite its lower toxicity, several cases have been reported with a range of serious complications, including neuropsychiatric sequelae, rhabdomyolysis resulting in acute kidney injury, ischemic and metabolic encephalopathy, ventricular fibrillation, multiorgan failure, and even death after exposure to imidacloprid [1–9].

We describe a case of suicidal imidacloprid ingestion without a classic toxidrome requiring ventilatory support and a prolonged neuropsychiatric sequela, and outline its management.

## Case presentation

**Fig. 1:**
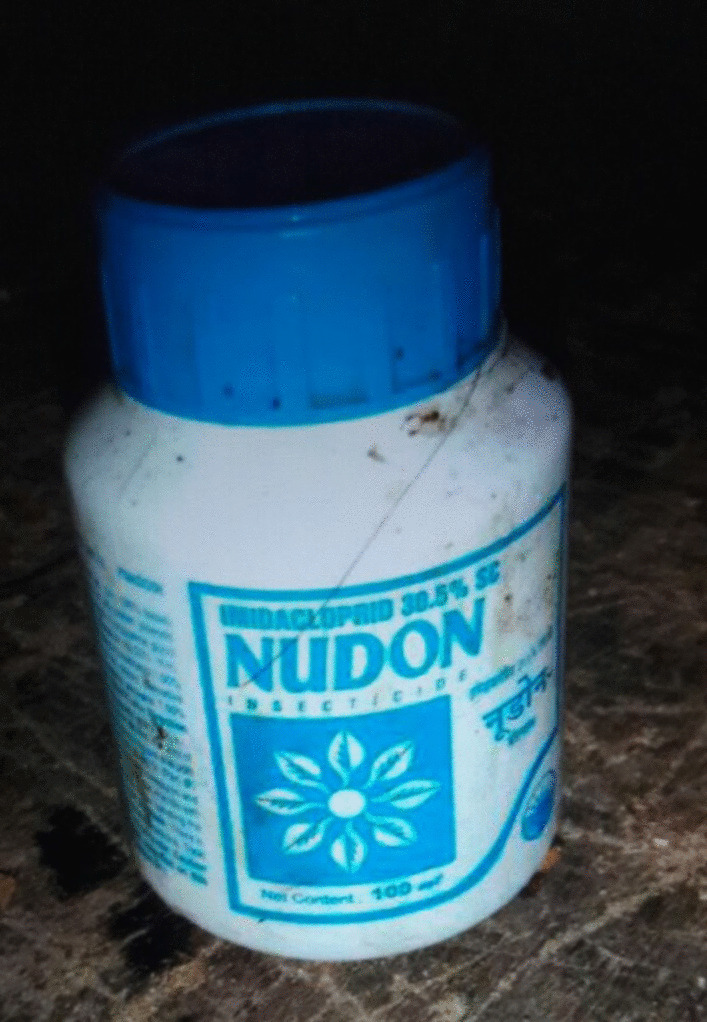
Image of involved poison

A 25-year-old previously healthy indigenous Tamang female was referred to our center following suicidal ingestion of an unknown amount of insecticide containing 30.5% imidacloprid (Nudon, India, Fig. 1) after appropriate initial management and gastric lavage with activated charcoal. On evaluation, she was disoriented [Glasgow Coma Scale (GCS) E4V4M4 12/15] with generalized fasciculations. She had a regular heart rate of 108 beats per minute, a blood pressure of 130/90 mmHg, respiratory rate of 20 breaths per minute; she was afebrile and was maintaining normal oxygen saturation at room air. Pupils were constricted, deep tendon reflexes were normal bilaterally, and bilateral crepitations were present on auscultation.

**Fig. 2:**
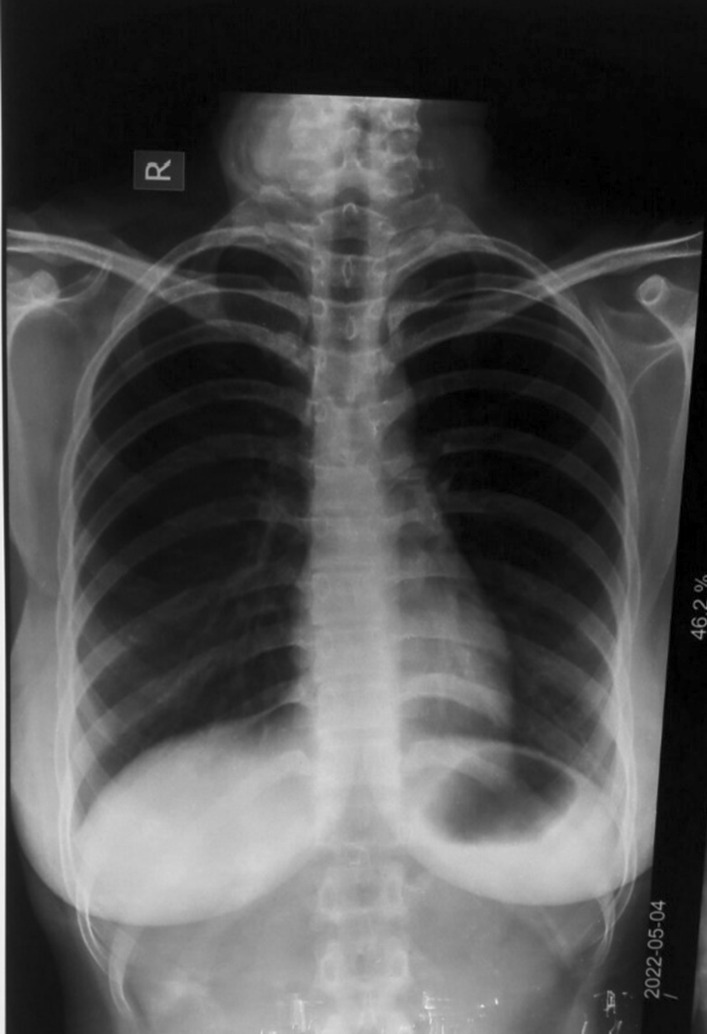
Chest x-ray showing lower lobe infiltrates

Unfortunately, her condition deteriorated rapidly, resulting in severe hypoxia. She was given one cycle of 0.6 mg atropine, was intubated and shifted to the intensive care unit (ICU), where she was mechanically ventilated. Her baseline electrocardiogram (ECG) and chest x-ray revealed no significant abnormalities, but 4 hours later, another chest x-ray revealed bilateral infiltrates in the lower lung fields (Fig. 2). Because of aspiration pneumonitis, intravenous antibiotics (intravenous clindamycin 600 mg TDS, intravenous piperacillin tazobactam 4.5 g TDS) were started. Initial arterial blood gas (ABG) showed metabolic acidosis with elevated lactate levels. However, it was not feasible to detect concentrations of imidacloprid in body fluids. A chronological record of the investigations is given in Table 1.

**Table 1:** Summary of laboratory investigations during hospital stay and their values

Laboratory findings of patient			
Test	Timing of the test	Value	Remarks
ABG	2 hours after ingestion	pH 7.26, pCO_2_ 31 mmHg, HCO_3_ 15.8 mmol/l, lactate 5.54 mmol/l	
	12 hours after ingestion	pH 7.4 pCO_2_ 32 mmHg, HCO_3_ 23.2 mmol/l, lactate 2.78 mmol/l	
	36 hours after ingestion	pH 7.53 pCO_2_ 24 mmHg, HCO_3_ 23.3 mmol/l, lactate 1.12 mmol/l	
	48 hours after ingestion	pH 7.53 pCO_2_ 24 mmHg, HCO_3_ 23.3 mmol/l, lactate 1.12 mmol/l	
Albumin/calcium	At admission	3.6 g/dl/8.8 mmol/l	
CBC	At admission	Hb 10 g%, TLC 13,200/cu mm N88% L5%	
	At discharge	Hb 10 g%, TLC 11,300/cu mm N86% L8%	
RFT	At admission	Na 146 mg/dl, K 3.15 mg/dl, urea 28 mg/dl, creatinine 0.8 mg/dl	
	At discharge	Na 134 mg/dl, K 4.20 mg/dl, urea 25 mg/dl, creatinine 0.9 mg/dl	
PT/INR	At admission	22 seconds/1.26	

*ABG* Arterial Blood Gas; *CBC* Complete Blood Count; *RFT* Renal Function Test; *PT/INR* Prothrombin Time /International Normalised Ratio

Supportive treatment was continued, which included prophylaxis against stress ulcers and deep venous thrombosis (DVT), and strict glycemic control was maintained. She developed hypotension on the second day of her ICU stay, which was managed with intravenous fluid boluses and vasopressors, and also had copious secretions, which were managed with glycopyrrolate. Her ICU stay was further complicated by an episode of generalized tonic–clonic seizure on the fourth day of admission, which was initially managed with injected lorazepam and then subsequently kept on intravenous levetiracetam 500 mg twice daily (BD) for 7 days.

After extubation, she remained hypoxemic and required oxygen supplementation for 7 days. On psychiatric evaluation during the ICU stay, the patient was delirious and was given a low dose of haloperidol (0.25 mg). After a prolonged and complicated stay, she was finally discharged on the tenth day of admission without any residual effects and was well oriented. A psychiatric reevaluation was done after the patient was medically stable, and she was diagnosed with a severe depressive episode. The patient and family members were counseled. Psychoeducation was given to both the patient and the patient’s family. She has been prescribed escitalopram 10 mg and is doing well on subsequent follow-ups.

## Discussion

Imidacloprid poisoning, although rarely fatal, is being increasingly reported from agricultural countries and is associated with some distinct neurological and other systemic findings [1, 4].

Neonicotinoids are nicotinic acetylcholine receptor (nAChR) agonists, inducing neuromuscular paralysis [7]. Lower toxicity can be explained based on the inherent structural differences between insect and mammalian nicotinic receptors [10].

Imidacloprid belongs to neonicotinoid compounds, and is the first neonicotinoid compound commercialized for widespread use. Based on animal studies, it is classified as moderately hazardous [Class-II World Health Organization (WHO); toxicity category-II US Environmental Protection Agency (EPA)] [1, 11].

It is chemically similar to nicotine, and other members of the neonicotinoid class include acetamiprid, clothianidin, thiacloprid, dinotefuran, nitenpyram, and thiamethoxam [11, 12]. These compounds can be absorbed via ingestion, dermal, or inhalation routes, and there is more severe poisoning with oral ingestion than with other routes. Neonicotinoids are agonists at nicotinic acetylcholine receptors and interfere with the transmission of impulses by increasing activation, leading to fatigue and paralysis. Receptor stimulation affects the Central Nervous System (CNS) as well as the autonomic nervous system [11, 13, 14].

Acute high-dose exposure in mammals primarily results in transient cholinergic effects (dizziness, apathy, locomotor effects, labored breathing), transient growth retardation, and even death. It may also be associated with cardiovascular and hematological effects, as well as degenerative changes in the testes, thymus, bone marrow, and pancreas [15].

Most of the cases are mild and may not come to clinical attention. Cases usually present with Gastrointestinal (GI) and neurological symptoms [8].

Neurological involvement may result in dizziness, drowsiness, disorientation, and coma, as well as features of autonomic nervous system stimulation. Autonomic stimulation may be associated with the risk of arrhythmia, hypotension, and bradycardia [1, 6, 7, 10].

Diagnosis is usually historical and can be aided by visual identification of the culprit poison. Some authors have described cases of toxicological analysis of biological fluids but with varied sensitivity and specificity [1, 2, 13].

As imidacloprid poisoning is associated with mild signs and symptoms, most cases are managed with close monitoring and symptomatic management. Patients developing respiratory compromise should be managed with invasive ventilation [1, 4, 14]. Previous case reports described a wide range of complications, from liver failure and rhabdomyolysis to death in some cases [1, 5–8]. Fatality might be related to aspirational pneumonia due to the rampant use of gastric lavage in emergency rooms, as well as due to co-ingestion of other toxins.

In a prospective study from Sri Lanka by Mohamed *et al*. comprising 68 patients with known imidacloprid poisoning, most patients only developed mild symptoms such as nausea, vomiting, headache, and diarrhea. No fatalities were reported, but one patient required mechanical ventilation due to respiratory failure. Patients rarely develop complications, the most serious being respiratory failure and a reduced level of consciousness [1].

Panigrahi *et al*. report a similar case of a patient with imidacloprid poisoning who developed respiratory arrest after about 20 hours of ingestion, requiring mechanical ventilation with subsequent recovery. Our case also had a similar clinical course, except for the earlier onset of respiratory failure and a prolonged neuropsychiatric sequel [16]].

## Conclusions

Although rarely reported in the medical literature, imidacloprid toxicity can occasionally manifest with life-threatening complications. It should be suspected in cases of unidentified poisoning with a toxidrome of acetylcholinergic symptoms and neurological involvement. Most patients improve with symptomatic management. A high level of clinical suspicion and close monitoring for the appearance of potential complications can help improve patient outcomes. A larger study could help better outline management principles.

## Data Availability

Not applicable.

## Abbreviations

GCS: Glasgow Coma Scale
ICU: Intensive care unit
ECG: Electrocardiography
TDS: Three times a day
DVT: Deep vein thrombosis
IV: Intravenous
ABG: Arterial blood gas
CBC: Complete blood count
RFT: Renal function test
PT/INR: Prothrombin time/International Normalized Ratio
TLC: Total leukocyte count
